# PYK2 negatively regulates the Hippo pathway in TNBC by stabilizing TAZ protein

**DOI:** 10.1038/s41419-018-1005-z

**Published:** 2018-09-24

**Authors:** Amir Kedan, Nandini Verma, Ashish Saroha, Michal Shreberk-Shaked, Anna-Katharina Müller, Nishanth Ulhas Nair, Sima Lev

**Affiliations:** 1 Molecular Cell Biology Department, Weizmann Institute of Science, Rehovot, 76100 Israel; 2 Center for Bioinformatics and Computational Biology, University of Maryland, College Park, MD 20742 USA

## Abstract

The tumor suppressor Hippo pathway negatively regulates the transcriptional coactivators Yes-associated protein (YAP) and transcriptional coactivator with PDZ-binding motif (TAZ) to inhibit cell growth and control organ size, whereas activation of YAP and TAZ is implicated in tumorigenesis and cancer metastasis. Here, we report that the nonreceptor tyrosine kinase PYK2 positively regulates TAZ and YAP transcriptional activity in triple-negative breast cancer (TNBC). We found that inhibition of PYK2 expression or its kinase activity substantially affects the steady-state level of TAZ and markedly facilitates its proteasomal degradation. This effect was specific to PYK2 inhibition and was not obtained by inhibition of FAK. Destabilization of TAZ was associated with profound effect of PYK2 inhibition on cell growth at low-density concomitant with reduced expression of TAZ-target genes and induction of cell apoptosis. We further show that PYK2 enhances the tyrosine phosphorylation of both TAZ and LATS1/2 and concomitantly TAZ stability, and that PYK2 protein level correlates with the level of TAZ protein in primary breast tumors. Together these observations suggest that PYK2 is an important regulator of the Hippo pathway, and its tyrosine kinase activity has a striking effect on TAZ stabilization and activation in TNBC.

## Introduction

The Hippo pathway is a highly conserved tumor suppressor cascade that regulates cell proliferation, apoptosis, and stem cell self-renewal to control organ cell numbers and size. The pathway is activated in response to different intrinsic signals such as cell–cell contact, cell adhesion, cell polarity, cell energy status, mechanical cues, and also in response to external hormonal signals^1,2^. Inactivation of the Hippo pathway is implicated in initiation and progression of multiple human tumors^3^.

The Hippo pathway is primarily propagated through activation of conserved Ser/Thr kinases including Hippo and Warts in *Drosophila* and their mammalian homologs MST1/2 and LATS1/2 (large tumor suppressor 1/2)^2^. Hippo and its binding partner Sav phosphorylate and activate Warts, which functions together with its regulatory subunit Mob to inhibit tissue growth^4^. The growth inhibitory effect of this kinase cascade is mainly mediated by inactivation of the transcriptional coactivator Yorkie in *Drosophila* and the two transcriptional coactivators, Yes-associated protein (YAP) and transcriptional coactivator with PDZ-binding motif (TAZ) in mammals.

Phosphorylation of YAP and TAZ by LATS1/2 prevents their nuclear translocation and consequently their association with TEA domain (TEAD) family of transcription factors^5,6^. YAP and TAZ also interact with RUNX^7^ and SMADS^8^ transcription factors to promote cell growth and survival. Hence, the Hippo pathway mainly imposes its tumor suppression activity through inhibition of YAP and TAZ, which are frequently activated in human cancers and have pleiotropic functions in tumor initiation and progression^3^.

YAP and TAZ share ~50% sequence identity and overall similar structural organization consisting of a PDZ domain, a TEAD-binding region, a coiled-coil domain and a WW domain that interacts with other proteins to control gene expression and cell fate^2^.

Previous studies showed that LATS1/2 phosphorylate YAP and TAZ on five and four serine residues, respectively^9,10^, and that phosphorylation of YAP at Ser127 and of TAZ at Ser89 promotes their binding to 14-3-3 proteins and consequently prevents their nuclear translocation. This cytoplasmic retention is accompanied by enhanced ubiquitination and their proteasomal degradation. Phosphorylation of TAZ at Ser311 and Ser314 by LATS1/2 and CK1ε, respectively, induces the formation of a C-terminal phosphodegron and the subsequent recruitment of the F-box protein β-TrCP and the SCF (Skip1, Cullin1, and F-box) E3 ubiquitin ligase complex for proteasomal degradation^11,12^. Importantly, TAZ contains additional N-terminal phosphodegron site^13^, and its degradation, as opposed to the cytoplasmic retention of YAP, appears to be the primary mode of TAZ inhibition^2^.

While the phosphorylation of YAP and TAZ on serine residues enhances their degradation, increasing line of evidence suggest that tyrosine phosphorylation stabilizes YAP and/or TAZ proteins. YAP, for example, is phosphorylated on Tyr357 by Yes1^14^, Src^15^, and by c-Abl in response to DNA damage^16^. Tyrosine phosphorylation of this site, which is located in close proximity to the YAP phosphodegron, stabilizes YAP^16^. Src also enhances the stability of TAZ. However, this stabilization is indirect and most likely mediated by tyrosine phosphorylation of LATS1, which inhibits LATS kinase activity^17^ as well as of β-TrCP, which attenuates the E3 Ubiquitin ligase activity of β-TrCP toward TAZ^18^.

Both YAP and TAZ are involved in cell proliferation, epithelial–mesenchymal transition, inhibition of apoptosis^19^, and are associated with aggressive tumor phenotype, cancer-stem cell features and metastasis^20,21^. Recent studies suggest that TAZ is highly expressed in breast cancer, in particular in the highly aggressive TNBC subtype^22–25^. Activation of TAZ has been correlated with high-histological grade, self-renewal of breast cancer-stem cells^20^, enhanced tumor metastasis, and poor outcome in breast cancer patients^26,27^. Hence, inhibition of YAP and/or TAZ activity and/or facilitating their degradation could have a therapeutic benefit for TNBC patients.

We previously showed that co-targeting of PYK2 and EGFR in basal-like TNBC cells inhibits cell proliferation in vitro and tumor growth in animal models^28^. Here, we show that inhibition of PYK2 expression or its tyrosine kinase activity robustly accelerates TAZ degradation in TNBC and consequently inhibits the expression of its target genes. We further show that PYK2 enhances the tyrosine phosphorylation of TAZ and LATS1/2 and stabilizes TAZ, and that PYK2, TAZ, LATS1/2, and β-TrCP can be found in the same immunocomplex. Hence, we propose that PYK2 negatively regulates the Hippo pathway to promote cell growth and prevent apoptotic cell death by stabilizing TAZ protein.

## Results

### PYK2 correlates with TAZ protein in TNBC and affects YAP and TAZ activation

**Fig. 1:**
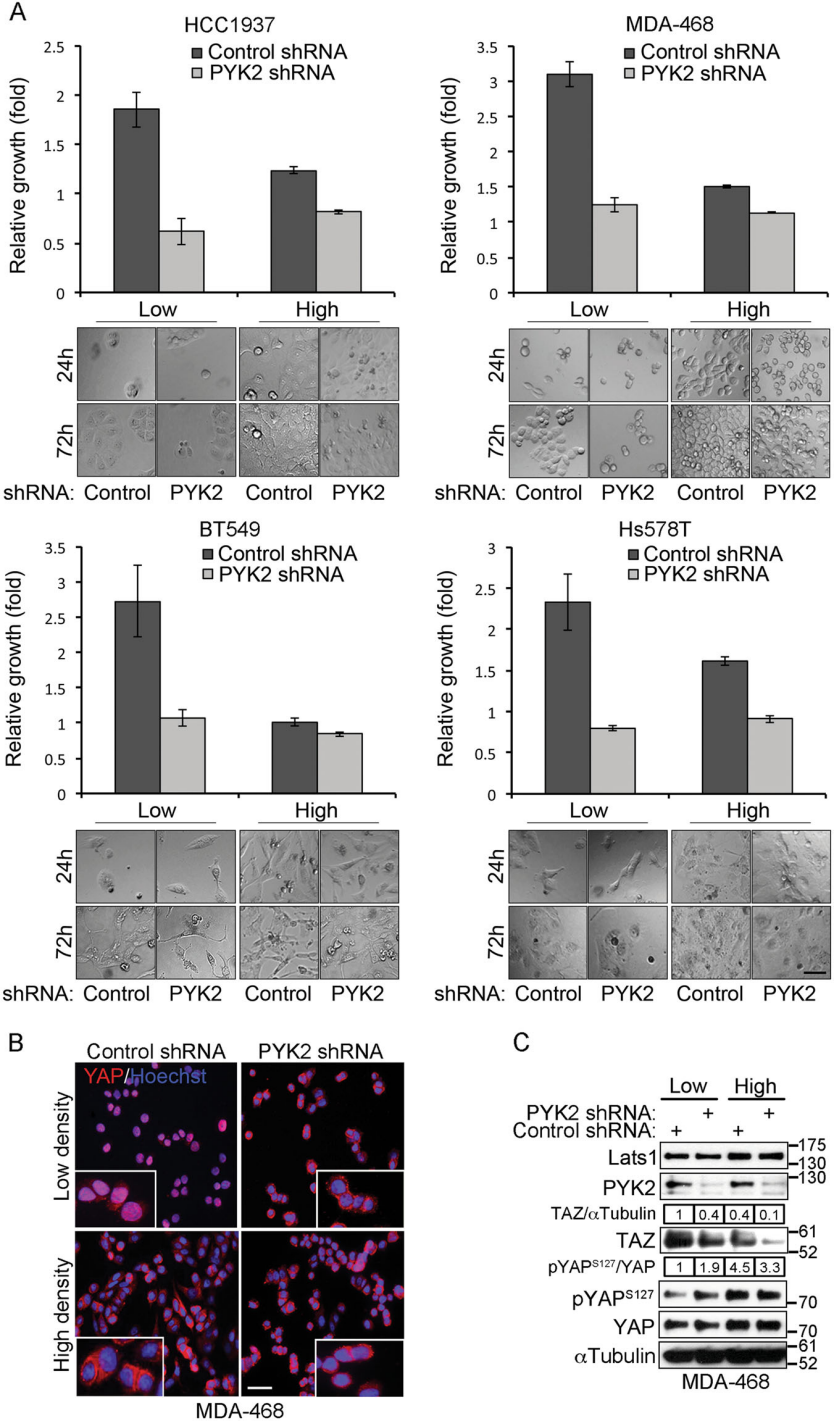
PYK2 depletion affects YAP/TAZ proteins and cell growth in a cell density-dependent manner. **a** The indicated control and PYK2-KD TNBC cell lines were seeded at low and high densities (Materials and methods) and cell viability at 24 and 72 h was determined by MTT assay. The ratio between cell viability at 72 and 24 h is shown (mean ± SD; *n* = 3) along with representative bright field microscope images of the cultured cells. Scale bar, 50 μm. **b** Subcellular localization of YAP in control and PYK2-KD MDA-468 cells grown at low and high densities, were examined by IF analysis. Scale bar, 50 μm. **c** Western blot (WB) analysis of TAZ protein level and YAP phosphorylation at Ser127 in control and PYK2-KD MDA-468 cells grown at low and high densities. Quantification is shown as fold of control (mean ± SD, *n* = 3)

We previously showed that depletion of PYK2 expression or inhibition of its kinase activity attenuates the proliferation of multiple basal-like TNBC cell lines^28^. More recently we observed that the effects of PYK2 depletion on cell growth were much more profound when cells were grown under sparse conditions compared to high-density culture (Figs. 1a and S1).

It is well-known that the Hippo pathway responds to contact inhibition and suppresses cell growth under high-cell density, whereas inactivation of the pathway under sparse conditions is associated with mitogenic response mediated by YAP/TAZ activation and the consequent induction of their target genes^9^.

**Fig. 2:**
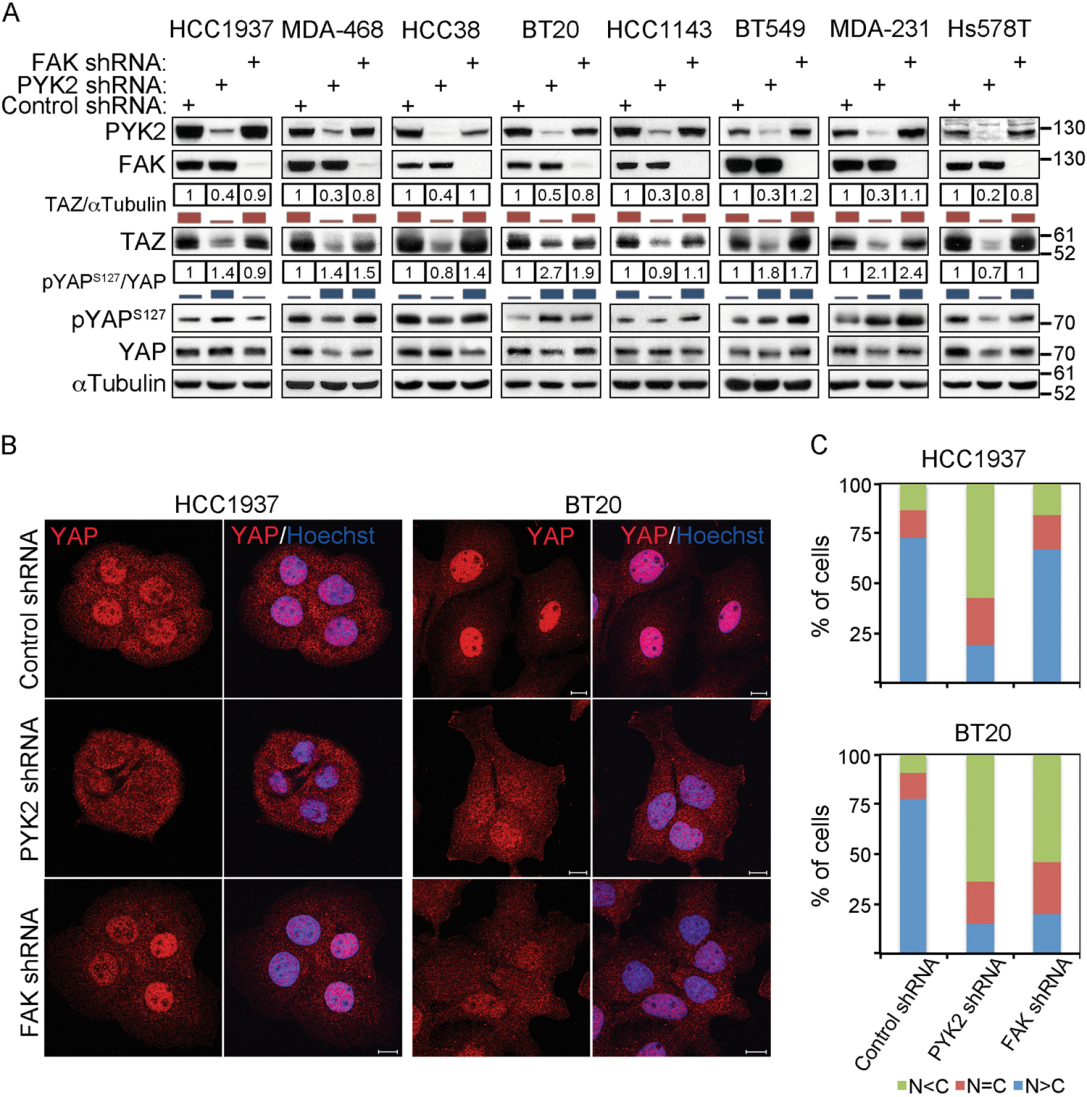
Depletion of PYK2 affects the protein level of TAZ and the Ser127 phosphorylation of YAP in TNBC cell lines. **a** WB analysis of TAZ protein level and YAP Ser127 phosphorylation in control and PYK2- or FAK-depleted TNBC cell lines. Quantification is shown as fold of control. **b** Subcellular localization of YAP in control and PYK2- or FAK-depleted TNBC cell lines (HCC1937, BT20), were examined by IF analysis. Scale bar, 10 μm. **c** The percentage of cells with nuclear (N) or cytosolic (C) YAP-staining was quantified based on the criteria shown below the graph (mean; *n* = 3)

The profound effect of PYK2 depletion on growth of sparse compared to dense cells implies that PYK2 depletion prevents the characteristic activation of YAP and/or TAZ. To explore this possibility, we examined the activation state and subcellular localization of YAP in control and PYK2-depleted MDA-MB-468 (MDA-468) cells grown under low and high-density conditions. The immunofluorescence (IF) images shown in Fig. 1b indicate that YAP was mainly localized in the nucleus of the control cells at low density and in the cytosol at high density, consistent with previous reports^9^. By contrast, YAP was localized in the cytosol of PYK2-depleted cells at both high and low densities, concurrent with an increase of its Ser127 phosphorylation (Fig. 1c). These results led us to examine the effect of PYK2 depletion on the activation states of YAP/TAZ in multiple TNBC cell lines. The cells were seeded at ~50% confluency and the activation of YAP/TAZ was assessed by western blotting (WB) and by IF analyses. As shown in Fig. 2a and S2A, knockdown (KD) of PYK2 expression by shRNAs or pools of PYK2 siRNAs markedly reduced the steady-state level of TAZ in multiple TNBC cell lines. The effect of PYK2 shRNA on TAZ protein level is shown in eight representative human TNBC cell lines of different molecular subtypes^29^. Importantly, expression of wild-type PYK2 in PYK2-depleted BT549 cells restored the expression of TAZ, thus demonstrating the specificity of PYK2 depletion on TAZ steady-state level (Fig. S2B). Furthermore, FAK depletion had no obvious effect on the level of TAZ protein. Together, these observations imply a correlation between PYK2 and TAZ protein level. Indeed, we found a significant positive correlation between the protein levels of PYK2 and TAZ (Fig. S2C, Spearman coefficient = 0.58, *P* = 0.002) in 27 primary breast cancer samples from TCGA, suggesting that PYK2 might regulate the abundance of TAZ in breast cancer patients.

In addition to the effect of PYK2 on TAZ, an increase in phospho-YAP(Ser127) was obtained in a subset of either PYK2- and/or FAK-depleted TNBC cell lines, which was accompanied with its cytoplasmic retention (Fig. 2b, c). These results suggest that PYK2 depletion has a robust effect on the steady-state level of TAZ, while PYK2 and/or FAK affect YAP activation in a cell-type dependent manner.

### PYK2 kinase activity is critical for activating YAP and stabilizing TAZ

**Fig. 3:**
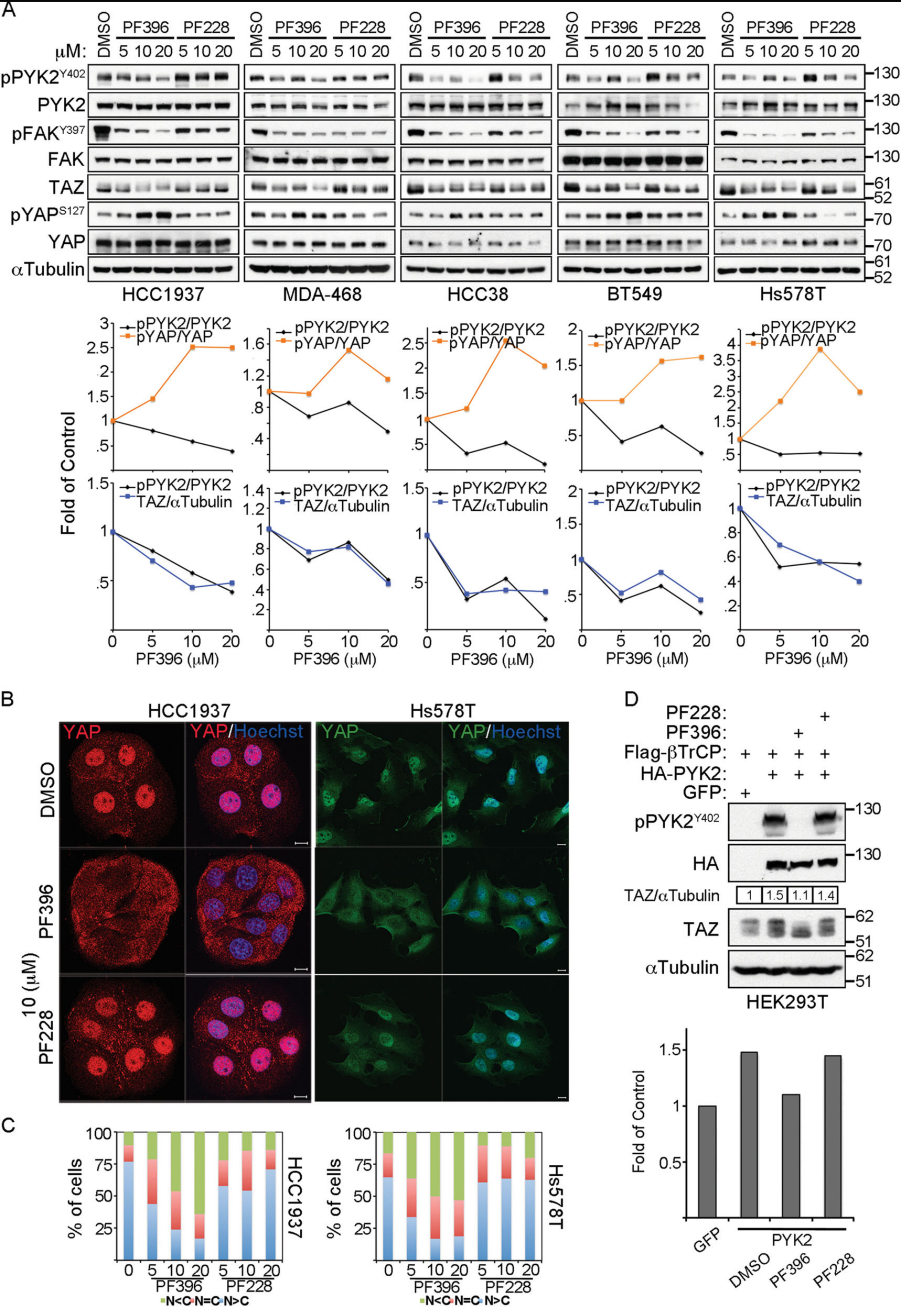
Inhibition of PYK2 kinase activity affects TAZ level and YAP Ser127 phosphorylation in TNBC cell lines. **a** The indicated TNBC cell lines were treated for 5 h with ascending concentrations of a dual PYK2/FAK (PF396) or a selective FAK (PF228) inhibitor. TAZ protein level and YAP Ser127 phosphorylation were determined by WB analysis. The correlating graphs show a quantified presentation of the WB results as fold of control. **b** Subcellular localization of YAP in cells treated as indicated was examined by IF analysis. Shown are representative results obtained in HCC1937 (YAP red) or Hs578T (YAP green) cells. Scale bar: 10 μm. **c** The percentage of cells with nuclear (N) or cytosolic (C) YAP-staining was quantified based on the criteria shown below the graph. (mean; *n* = 3). **d** HEK293T cells were transfected with HA-PYK2 and its influence on endogenous TAZ was determined in the presence or absence of PYK2/FAK inhibitors. Quantification shown below in bar graphs as fold of control

To assess the influence of PYK2 and/or FAK kinase activity on the steady-state level of TAZ and the phosphorylation of YAP at Ser127, we used two commercially available inhibitors: PF573228 (PF228), a selective FAK inhibitor^30^, and PF431396 (PF396), a dual PYK2/FAK inhibitor^31^. As seen in Fig. 3a, while FAK inhibitor had no significant effects on the steady-state level of TAZ, the dual PYK2/FAK inhibitor markedly reduced the steady-state level of TAZ. The effect was dose-dependent and coupled to PYK2 activation as assessed by its Tyr402 phosphorylation. YAP Ser127 phosphorylation was also increased in response to PF396 treatment, consistent with its cytosolic localization shown by the IF analysis (Fig. 3b, c).

To further demonstrate the effect of PYK2 kinase activity on TAZ stability, we expressed PYK2 in HEK293T cells and examined its influence on TAZ steady-state level in the absence or presence of FAK inhibitor or the PYK2/FAK dual inhibitor. As shown, overexpression of PYK2 in HEK293T cells increased the level of TAZ protein and the dual PYK2/FAK kinase inhibitor (PF396) abolished this effect while FAK inhibitor (PF228) had no effect (Fig. 3d). Collectively, these results suggest that inhibition of PYK2 kinase activity markedly reduced the protein level of TAZ in TNBC cells and also increased YAP phosphorylation at Ser127.

### PYK2 inhibition facilitates the proteasomal degradation of TAZ

**Fig. 4:**
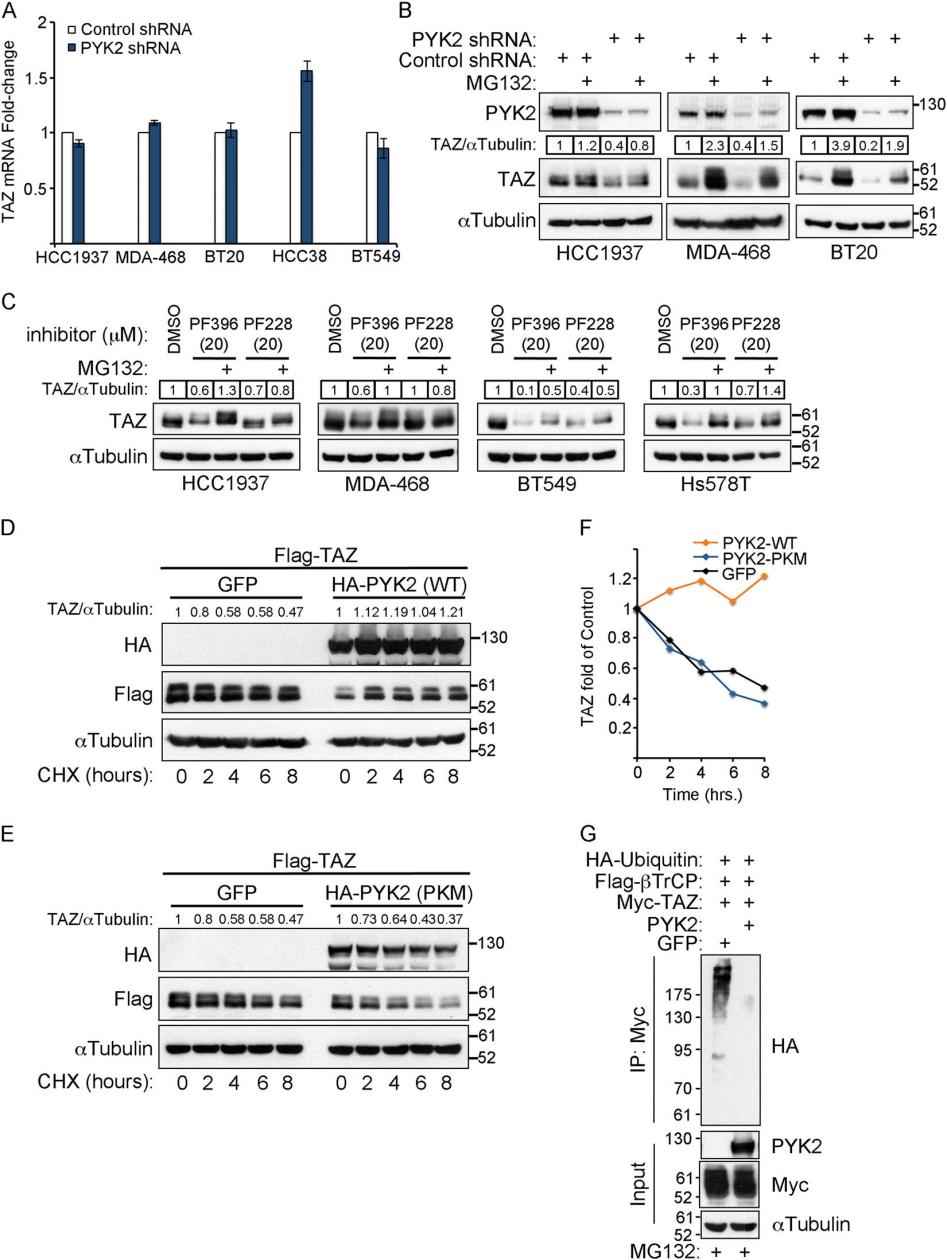
PYK2 attenuates the proteasomal degradation of TAZ. **a** qRT-PCR analysis of TAZ mRNA expression in control and PYK2-KD TNBC cells. (mean ± SD; *n* = 3). **b** The level of TAZ protein in control and PYK2-KD TNBC cell lines was determined by WB analysis in the absence or presence of MG132 (5 μM for 16 h for all lines except for MDA-468, 1 μM). Quantification is shown as fold of control. **c** The indicated TNBC cell lines were treated with the different concentrations of the FAK (PF228) or the dual PYK2/FAK inhibitor (PF396) for 5 h in the presence or absence of MG132 (5 μM) and then analyzed by WB. Quantification is shown as fold of control. **d**, **e** HEK293T cells coexpressing Flag-TAZ with either the wild-type PYK2 (PYK2-WT) (**d**) or its kinase deficient mutant, PKM (**e**) were incubated with 50 μg ml^−1^ cycloheximide for the indicated time points. The levels of Flag-TAZ were determined by WB analysis. Quantification is shown as fold of time 0. **f** Quantification of the WB results shown in **d** and **e**. **g** HEK293T cells expressing HA-ubiquitin, Myc-TAZ, Flag-β-TrCP and either PYK2 or GFP as indicated were treated with 10 μM MG132 for 5 h. The cells were then lysed, and Myc-TAZ-associated ubiquitination was assessed by WB of immunoprecipitated Myc-TAZ with anti-HA antibody

As PYK2 depletion/inhibition markedly affects the steady-state level of TAZ in multiple TNBC cell lines (Figs. 2 and 3), it might affect the transcription level of TAZ and/or its protein stability. The mRNA levels of TAZ in control and PYK2-depleted TNBC cells were assessed by quantitative reverse transcription polymerase chain reaction (qRT-PCR), and as shown, the levels were similar (Fig. 4a), implying that PYK2 affects TAZ protein level.

TAZ is an extremely unstable protein that is degraded by the proteasome^11,12^, therefore, MG132, a proteasomal degradation inhibitor, was used to examine the effect of PYK2 on TAZ degradation. As shown in Fig. 4b, MG132 increased the steady-state level of TAZ in both control and PYK2-depleted cells, but the effects were more profound in PYK2-depleted cells and could restore TAZ levels of control cells. Likewise, MG132 almost completely abolished the effect of the dual PYK2/FAK inhibitor (PF396) on TAZ protein level (Fig. 4c), suggesting that PYK2 depletion/inhibition enhances the proteasomal degradation of TAZ. It is worth mentioning that the effect of MG132 on the steady-state level of TAZ varied between the different TNBC cell lines, possibly due to their PTEN/PI3K status which is known to influence TAZ stability^12,13^. Among the different lines, BT549, HCC1937, and MDA-468 are PTEN negative, while BT20 is PI3KA positive^32^.

The profound effect of PYK2 depletion/inhibition on TAZ degradation implies that PYK2 stabilizes TAZ. To test this possibility, PYK2 (Fig. 4d) or its kinase deficient mutant PKM (Fig. 4e) were overexpressed in HEK293T cells together with Flag-TAZ, and TAZ degradation over time was examined in the presence of cycloheximide, a protein synthesis inhibitor. As shown in Fig. 4d–f, while the wild-type PYK2 inhibited the degradation of Flag-TAZ, PKM had no effect on TAZ degradation, suggesting that the kinase activity of PYK2 is required for TAZ stabilization.

Previous studies have shown that the proteasomal degradation of YAP/TAZ is mediated by the recruitment of the F-box protein β-TrCP to their phosphodegrons and their subsequent ubiquitination by the E3 SCF^β-TrCP^ ligase complex^11,12^. Hence, we examined the ubiquitination of TAZ by β-TrCP in the presence and absence of PYK2. Myc-TAZ was coexpressed with Flag-β-TrCP, HA-ubiquitin and either GFP or PYK2, in HEK293T cells. Myc-TAZ was immunoprecipitated from MG132-treated cells and its ubiquitination was assessed by immunoblotting with anti-HA antibody. As shown in Fig. 4g, the ubiquitination of TAZ was markedly reduced in the presence of PYK2, further suggesting that PYK2 stabilizes TAZ.

### Inhibition of GSK-3β or LATS1/2 partially restored TAZ stability in PYK2-depleted cells

**Fig. 5:**
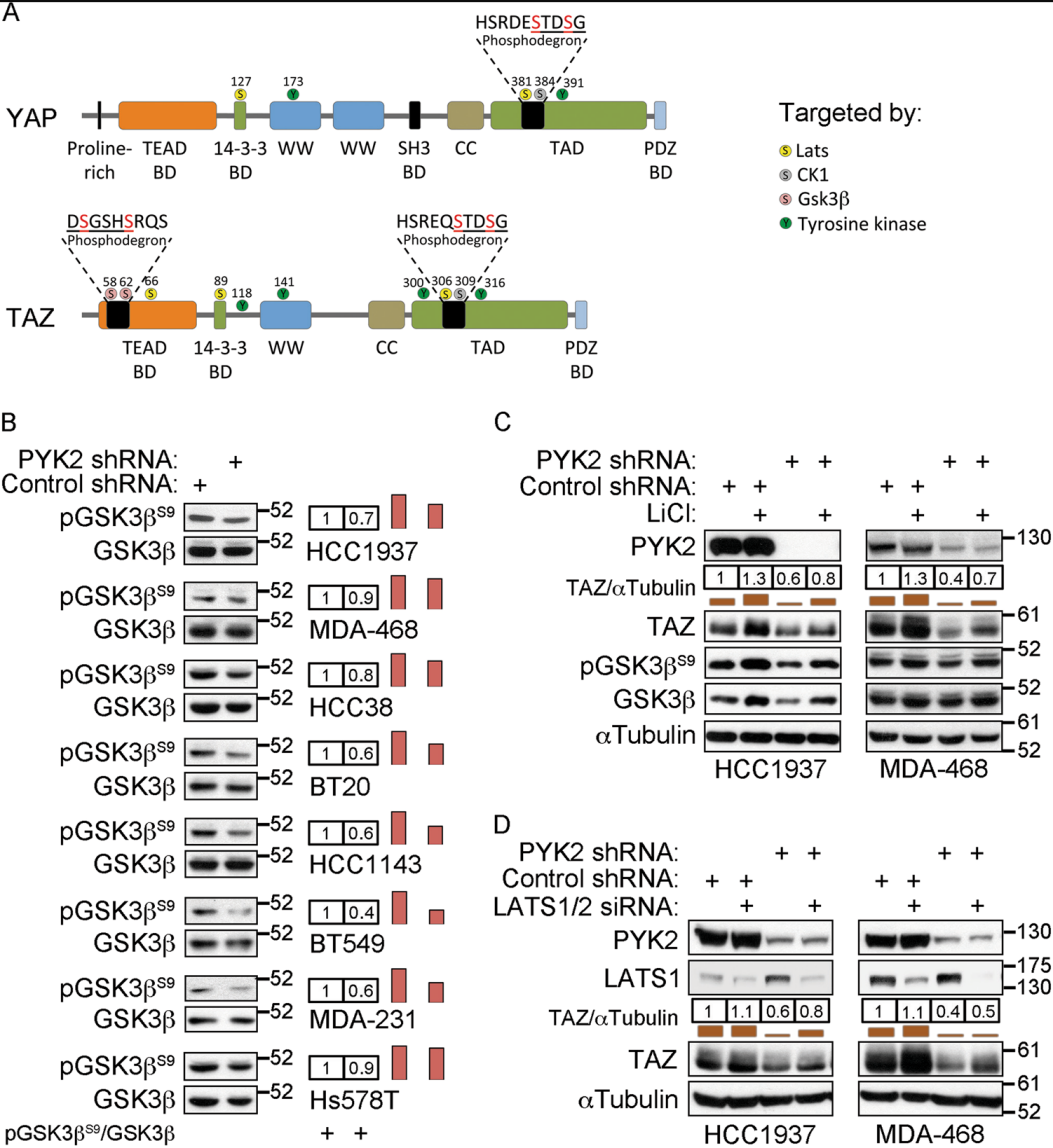
GSK-3β inhibition or LATS1/2 depletion partially restored the level of TAZ in PYK2-depleted cells. **a** Schematic presentation depicting the domains of mYAP and mTAZ, and the residues targeted by post-translational modifications. The serine residues that are targeted by LATS1/2, CK1ε, and GSK-3β are shown in yellow, gray, and pink, respectively, and the tyrosine phosphorylation sites are shown in green. BD binding domain; CC coiled-coil; TAD transactivation domain. **b** The phosphorylation of GSK-3β at Ser9 in control and PYK2-depleted TNBC cell lines was determined by WB analysis. The bands intensities were quantified and results are shown as fold of control. **c** Control and PYK2-depleted TNBC cell lines were treated with 10 mM LiCl for 16 h and then analyzed by WB for TAZ protein level. Quantification is shown as fold of control. **d** Control and PYK2-KD TNBC cell lines were transfected with either scrambled or LATS1/2 siRNA and then analyzed by WB for TAZ protein level. Quantification is shown as fold of control

Previous studies have shown that TAZ contains two consensus phosphodegron motifs for β-TrCP binding; one at its N-terminal and the second at its C-terminal region (Fig. 5a)^12,13^. The C-terminal, but not the N-terminal one, is conserved in YAP^33^. The N-terminal phosphodegron was proposed to recruit β-TrCP in response to TAZ phosphorylation at Ser58/Ser62 by GSK-3β^13^, while the C-terminal was proposed to be phosphorylated by LATS1/2 and CK1ε^11,12^.

To examine whether GSK-3β activation is involved in the degradation of TAZ in PYK2-KD cells, we first assessed its activation in control and PYK2-depleted TNBC cells. GSK-3β activity is negatively regulated by PI3K/AKT-mediated phosphorylation of its Ser9^34^. Hence, the level of GSK-3β-Ser9 phosphorylation in control and PYK2-depleted TNBC cells was used to monitor GSK-3β activity. As shown in Fig. 5b, PYK2 depletion led to a slight-to-moderate (0.9–0.4-fold) decrease in GSK-3β-Ser9 phosphorylation, suggesting that PYK2 depletion slightly enhances GSK-3β activity. We then examined whether inhibition of GSK-3β by LiCl (Figs. 5c and S3A) BIO-X or SB216763 (Figs. S3B, C, respectively) could restore the level of TAZ in PYK2-depleted cells, and found only a partial effect. Likewise, depletion of LATS1/2, which phosphorylate the C-terminal phosphodegron^11,12^, could partially restore TAZ level in a subset of PYK2-depleted TNBC cells (Fig. 5d). Furthermore, depletion of LATS1/2 together with inhibition of GSK-3β by LiCl partially restored the level of TAZ in a cell-specific manner (Fig. S3D), implying that PYK2 depletion may enhance the phosphorylation of both the N- and C-terminal phosphodegron sites and/or destabilize TAZ via additional mechanisms.

### PYK2 enhances tyrosine phosphorylation of TAZ and LATS1/2

**Fig. 6:**
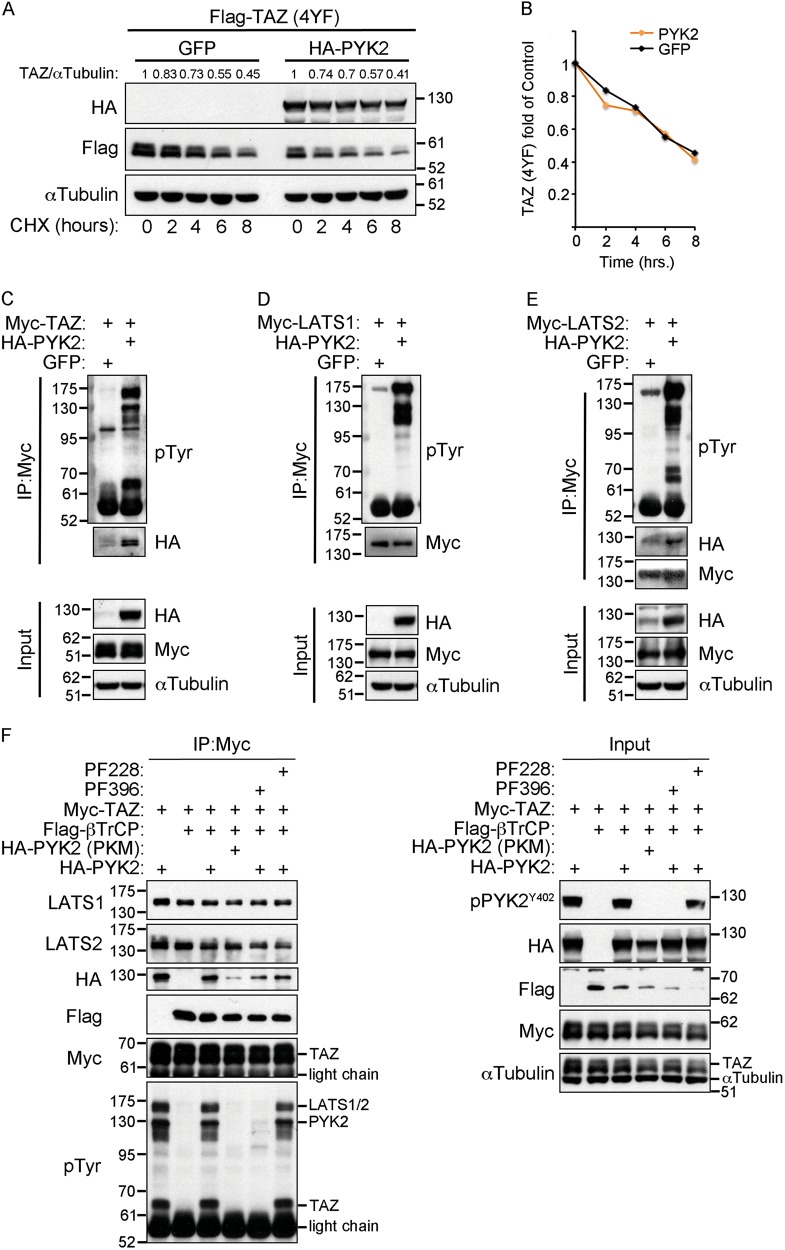
PYK2 enhances tyrosine phosphorylation of TAZ and LATS1/2 but has no effect on degradation of TAZ(4YF) mutant. **a** HEK293T cells were transfected with the indicated DNA constructs and 36 h later treated with 50 μg/ml cycloheximide for the indicated time points. The levels of Flag-TAZ were determined by WB analysis. **b** Quantification is shown as fold of time 0. **c**–**e** HEK293T cells were cotransfected with HA-PYK2 and Myc-TAZ (**c**), Myc-LATS1 (**d**), or Myc-LATS2 (**e**). Myc-tagged LATS1/2 or TAZ were immunoprecipitated by anti-Myc antibody and their tyrosine phosphorylation was determined by WB using antiphosphotyrosine antibody (pTyr). **f** HEK293T cells were transfected with the indicated DNA constructs and treated with 5 μM MG132 for 5 h. Myc-TAZ was immunoprecipitated by anti-Myc antibody and the presence of endogenous LATS1/2, Flag-β-TrCP or HA-PYK2 in Myc-TAZ immunocomplex was determined by WB analysis using the corresponding antibodies. Tyrosine phosphorylated proteins associated with TAZ-immunocomplex were detected by antiphosphotyrosine antibody

Both YAP and TAZ undergo tyrosine phosphorylation by the nonreceptor tyrosine kinase c-Abl, which enhances their stabilization and consequently the transcription of specific target genes^16,35^. The profound effect of PYK2 on TAZ stability (Figs. 2 and 3) led us to examine whether PYK2 stabilizes TAZ via tyrosine phosphorylation. We first assessed the effect of PYK2 expression on the degradation of TAZ mutant lacking its four tyrosine residues (TAZ-4YF) sites^18^, using the cycloheximide chase experiment described in Fig. 4d. As shown, PYK2 had no effect on the degradation of the TAZ-4YF mutant (Fig. 6a, b), while stabilizing the wild-type TAZ protein (Fig. 4c, f), implying that the effect of PYK2 is mediated by tyrosine phosphorylation of TAZ.

To test this possibility, we coexpressed Myc-TAZ in HEK293T cells either with GFP or with PYK2, immunoprecipitated Myc-TAZ and examined its tyrosine phosphorylation by WB. As shown, TAZ was strongly phosphorylated on tyrosine residues in the presence of PYK2 (Fig. 6c). We further detected two additional high molecular weight tyrosine phosphorylated proteins in TAZ-immunocomplex; one in PYK2 size (~130 kDa) and another of ~160 kDa, resembling the size of LATS1/2.

It was shown that Src phosphorylates LATS1 on tyrosine residues and consequently inhibits its activity^17^. To test whether PYK2 also induces tyrosine phosphorylation of LATS1/2, we immunoprecipitated LATS1/2 from control HEK293T cells and cells that overexpress PYK2. As shown in Fig. 6d, e, tyrosine phosphorylation of LATS1/2 was strongly induced in the presence of PYK2, suggesting that PYK2 enhances the tyrosine phosphorylation of both TAZ and LATS1/2. Furthermore, the three proteins were detected in the same immunocomplex, and thus, may interact with each other (Fig. 6c). As TAZ interacts with β-TrCP and β-TrCP was proposed to be tyrosine phosphorylated by Src^18^, we assessed both the tyrosine phosphorylation and the interaction between PYK2, TAZ, β-TrCP, and LATS1/2 by a coimmunoprecipitation experiment. HEK293T cells were cotransfected with Flag-β-TrCP, Myc-TAZ, and either HA-tagged PYK2 or PKM. The cells were treated with FAK or the dual PYK2/FAK inhibitors to demonstrate the influence of PYK2 kinase activity. As shown in Fig. 6f, TAZ as well as endogenous LATS1/2 were tyrosine phosphorylated only in the presence of active PYK2, and tyrosine phosphorylated TAZ, LATS1/2, and PYK2 were found in the same immunocomplex. Importantly, in contrast to activated Src, which enhances the tyrosine phosphorylation of β-TrCP^18^, PYK2 had no detectable effect on the tyrosine phosphorylation of β-TrCP. Furthermore, TAZ had a stronger interaction with the kinase-active PYK2. These results suggest that TAZ is a possible PYK2 substrate, whereas LATS1/2 might be directly phosphorylated by PYK2 or indirectly through activation of Src^36^. Collectively, we show that PYK2 induces tyrosine phosphorylation of key components of the Hippo pathway and positively regulates TAZ stability in its kinase activity-dependent manner.

### PYK2 depletion inhibits YAP/TAZ coactivation and induces apoptosis

**Fig. 7:**
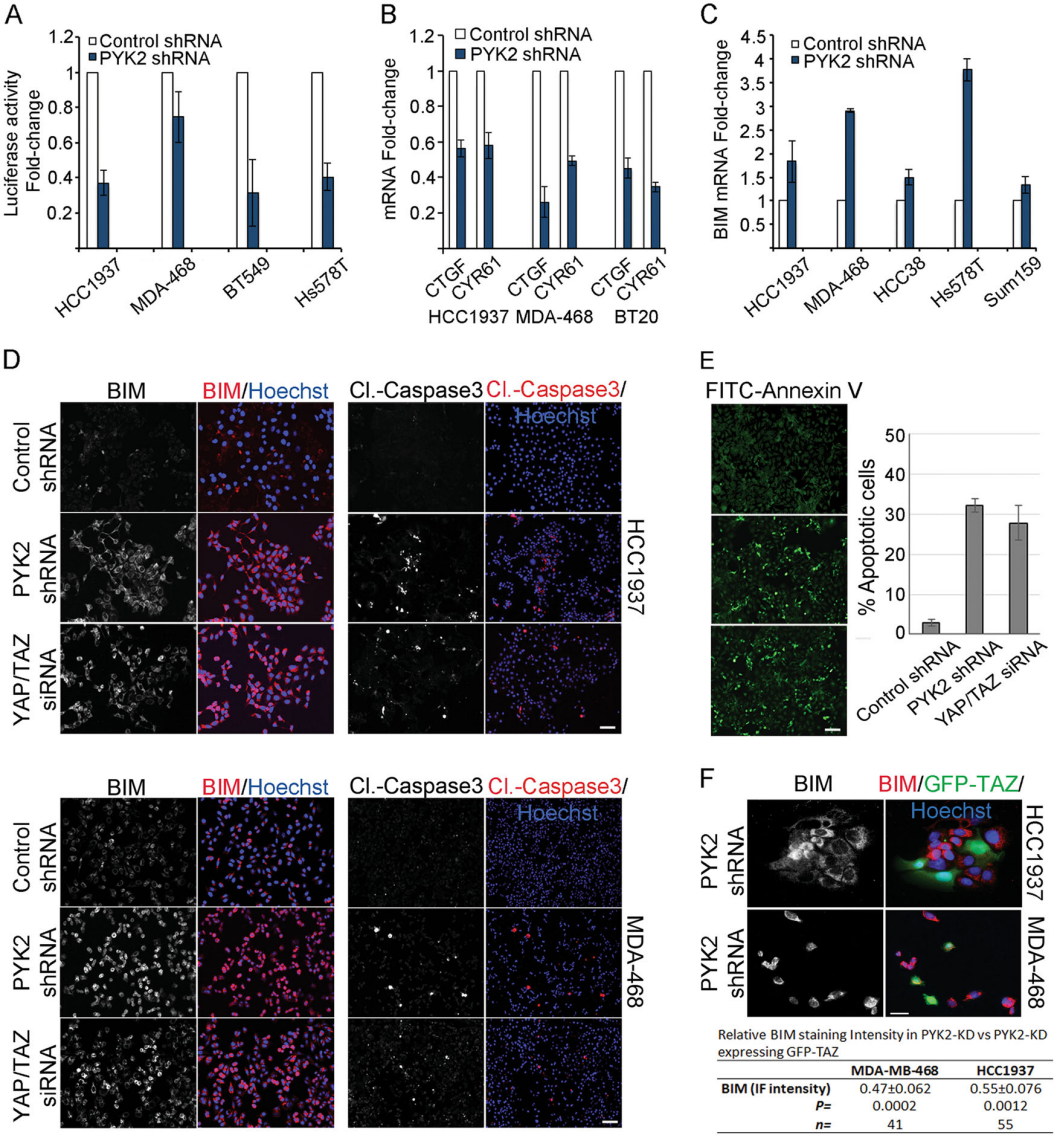
Effects of PYK2 depletion on YAP and TAZ-downstream targets and on apoptosis. **a** Control and PYK2-KD TNBC cell lines were transfected with the GTIIC-luciferase reporter and luciferase activity was measured as described in Methods (mean ± SD; *n* = 2). **b** Bar graphs showing qRT-PCR analysis of YAP/TAZ specific targets (CYR61 and CTGF) in control and PYK2-KD TNBC cells. (mean ± SD; *n* = 2). **c** BIM mRNA expression was estimated by qRT-PCR analysis in control and PYK2-KD TNBC cells. (mean ± SD; *n* = 2). **d** IF staining micrograph panels showing BIM and cleaved caspase-3 staining in control, PYK2-KD and YAP/TAZ-KD HCC1937 and MDA-468 cells. **e** FITC-Annexin V staining assay was performed to detect apoptotic cells in control, PYK2-KD and YAP/TAZ-KD HCC1937 cells as described in Materials and methods. Bar graph representing the % of apoptotic cells calculated by counting the number of FITC-Annexin V positive cells in control and experimental panels (five microscopic fields taken from three different experimental replicates). **f** IF staining of BIM (red) in PYK2-depleted HCC1937 and MDA-468 cells expressing GFP-TAZ (green). The calculated ratios (lower table) represent the relative intensity of BIM IF staining in PYK2-KD cells expressing GFP-TAZ compared to BIM intensity in PYK2-KD cells. Quantitation was performed as described in Materials and Methods and mean values ± SD of BIM intensity in ~45 cells from three independent experimental replicates are shown. Scale bar, 50 μm

The profound effect of PYK2 on TAZ stability in multiple TNBC cell lines suggests that PYK2 affects YAP/TAZ-downstream targets. This was demonstrated by a luciferase reporter assay using the GTIIC-luciferase reporter (Fig. 7a) and the expression of direct YAP/TAZ-target genes (CTGF and CYR61) by qRT-PCR (Fig. 7b). The GTIIC-luciferase reporter contains TEAD-binding element-driven luciferase and is commonly used to measure activation of the Hippo pathway. As shown, depletion of PYK2 reduced the luciferase activity as well as CTGF and CYR61 transcription, indicating that PYK2 depletion inhibits the expression of YAP/TAZ-target genes.

Previous studies suggest that activation of YAP/TAZ inhibits apoptosis partially through suppression of BIM, a proapoptotic protein^37^. Indeed, qRT-PCR analysis indicated that KD of PYK2 elevated the transcription of BIM (Fig. 7c). Moreover, reverse phase protein array (RPPA) analysis of multiple TNBC cell lines depleted of PYK2 showed profound effects of PYK2-KD on BIM protein levels (Fig. S4A). As PYK2-KD enhanced TAZ degradation and consequently suppressed the expression of YAP/TAZ-target genes, it could be that YAP/TAZ inhibition mediates, at least partially, the effect of PYK2-KD on BIM expression and apoptotic cell death. To explore this possibility, we examined the level of BIM and cleaved casapase-3 in control, PYK2-KD or YAP/TAZ-depleted HCC1937 and MDA-468 cells (Fig. 7d) by IF analysis. As shown, KD of PYK2 markedly enhanced BIM expression and caspase-3 cleavage in both cell lines. Similarly, depletion of YAP/TAZ by siRNA had strong effects on BIM expression and caspase-3 cleavage, suggesting that depletion of PYK2 or YAP/TAZ enhances BIM protein level in these cells and concomitantly apoptotic cell death. Indeed, Annexin V staining of PYK2- or YAP/TAZ-depleted HCC1937 cells showed a significant increase in FITC-Annexin V positive cells (~30%) (Fig. 7e), and PYK2 or YAP/TAZ silencing markedly increased the level of cleaved PARP in HCC1937 and MDA-468 (Fig. S4B, C).

We then examined whether YAP/TAZ inhibition mediates the proapoptotic effects of PYK2 depletion by overexpressing TAZ in PYK2-KD MDA-468 and HCC1937 cells. The results shown in Fig. 7f indicate that ectopic expression of GFP-TAZ in PYK2-KD cells significantly decreased the expression of BIM (by ~50%) and thus partially rescued the effect of PYK2-KD on BIM upregulation. These results suggest that PYK2 inhibition enhances apoptosis, at least partially, by destabilizing TAZ.

## Discussion

The Hippo pathway suppresses the activity of YAP/TAZ and controls cell growth and organ size to maintain tissue homeostasis^1^. Inactivation of this pathway, and thus activation of YAP/TAZ, leads to aberrant cell growth, cell transformation and tumorigenesis^3^. YAP/TAZ activity is tightly regulated and controlled by multiple mechanisms that either promote or prevent their nuclear translocation, transcriptional coactivation, and/or proteasomal degradation^9,11,12^. YAP is a relatively stable protein, predominantly regulated by phosphorylation, and thus, by its cytosol-to-nucleus shuttling, whereas TAZ, an extremely unstable protein, is primarily regulated by protein degradation^2^.

Here we show that depletion of PYK2 expression or inhibition of its kinase activity robustly accelerated the proteasomal degradation of TAZ in multiple TNBC cell lines (Figs. 2a and 3a), and consequently reduced the transcription of YAP/TAZ-target genes (Fig. 7a–c). The effect of PYK2 on TAZ degradation was specific to PYK2 as it was not obtained by FAK inhibition, although both FAK and PYK2 had some effects on YAP Ser127 phosphorylation (Fig. 2a). Strikingly, we recently showed that inhibition/depletion of PYK2 also facilitates the degradation of HER3, and that inhibition of PYK2 not only sensitized basal-like TNBC to EGFR antagonists but could also circumvent HER3-associated drug resistance^28^. Consistent with our findings, a previous report showed that TAZ KD sensitized TNBC cells to EGFR antagonists^38^. Hence, it could be that PYK2 inhibition   sensitized basal-like TNBC to EGFR antagonists, at least in part, by destabilizing TAZ.

The robust effects of PYK2 inhibition on TAZ degradation (Fig. 3a), concomitant with the effect of overexpresion of kinase-active PYK2, on TAZ stability (Fig. 3d), suggest that PYK2 stabilizes TAZ in its kinase activity-dependent manner. The stability of TAZ is regulated by both tyrosine and serine phosphorylation. GSK-3β was proposed to enhance TAZ degradation by phosphorylating TAZ on two N-terminal phosphodegron sites (Fig. 5a) in response to PI3K/AKT pathway inhibition^13^, or alternatively by phosphorylating β-catenin and recruiting TAZ to β-TrCP^39^. Interestingly, we found that depletion of PYK2 in several TNBC lines reduced GSK-3β phosphorylation on Ser9 (Fig. 5b), and thus may enhance its activity. Indeed, we could partially restore the level of TAZ in PYK2-depleted cells by inhibition of GSK-3β (Fig. 5c, S3A–C), and although previous reports showed that PYK2 induced tyrosine phosphorylation of GSK-3β at Tyr216^40^, currently the role of this phosphorylation remains controversial^41^.

In addition to the N-terminal phosphodegron, both YAP and TAZ have a C-terminal phosphodegron, which is phosphorylated by LATS1/2 and CK1ε^12^. Depletion of LATS1/2 partially restored TAZ level in PYK2-KD cells (Fig. 5d), suggesting that additional mechanisms are involved. We propose that LATS1/2 inhibition, GSK-3β activation (Fig. 5b), and TAZ tyrosine phosphorylation (Fig. 6c) are affected by PYK2 tyrosine kinase activity. Since PYK2 enhanced the tyrosine phosphorylation of LATS1/2 (Fig. 6d, e), it could be that it phosphorylates LATS1/2 directly or indirectly via Src. Indeed, previous studies showed that Src phosphorylates LATS1 and inhibits its activity^17^, and that Src attenuates the activity of β-TrCP towards TAZ, independently of TAZ tyrosine phosphorylation^18^. Our results suggest that PYK2 enhances TAZ tyrosine phosphorylation and concomitantly stabilizes TAZ. Hence, it could be that PYK2 cooperates with Src to profoundly stabilize TAZ and prevent its degradation, and/or that PYK2 and Src employ complementary mechanisms to ensure stabilization of TAZ and transcription of its target genes. Nevertheless, further studies are needed to decipher the exact mechanistic role of PYK2 on TAZ and the Hippo pathway.

In light of our findings and of previous reports, key components of the Hippo pathway including LATS1/2, YAP and TAZ undergo tyrosine phosphorylation, which regulate their function. Tyrosine phosphorylation of YAP by c-Abl at Tyr391 affects its stability and activity in response to DNA damage^16^. The homologs site in TAZ, Tyr316, is phosphorylated by c-Abl upon hyperosmotic stress to suppress the transcription factor NFAT5^35^. These tyrosine residues are located at the transcription activation domains and in the vicinity of their phosphodegrons (Fig. 5a). Moreover, Yes phosphorylates YAP, increases its association with TEAD and consequently the activation of Oct3/4 and Nanog^14^, suggesting that tyrosine phosphorylation of YAP and TAZ influences the selectivity of their target genes. Interestingly, a recent study suggests that tyrosine phosphorylation of YAP at Tyr188 (Tyr173 in mouse) within its WW domain affects YAP oncogenic functions, possibly by interfering YAP–LATS1 interactions^42^. We could also detect interaction between LATS1/2 and TAZ by coimmunoprecipitation and found PYK2 in the same immunocomplex (Fig. 6f), implying that the three proteins can interact with each other. In addition, we observed that KD of PYK2 affects the expression of YAP/TAZ-target genes (Fig. 7a, b) including the expression of the proapoptotic protein BIM (Fig. 7c). Finally, we showed that depletion of PYK2, as well as of YAP/TAZ, enhanced the expression of BIM, and concurrently the cleavage of caspase-3 (Fig. 7d) and PARP (Fig. S4B, C), and that the proapoptotic effects of PYK2-KD are partially mediated by TAZ (Fig. 7f).

In summary, our findings demonstrate that PYK2 has a profound effect on TAZ stability and thus regulates the Hippo pathway. PYK2, through its tyrosine kinase activity increases the tyrosine phosphorylation of LATS1/2 and TAZ, stabilizes TAZ, and enhances TAZ-regulated cellular processes including cell survival, growth and apoptosis. We propose that PYK2 positively regulates the high level of TAZ protein in TNBC, which is associated with high histological grade and poor clinical outcome.

## Materials and methods

### Antibodies, reagents, and chemicals

The following antibodies were purchased from Santa Cruz Biotechnology (Santa Cruz, CA): pPYK2 (Y402, sc-101790), FAK (sc-558), YAP/TAZ (63.7, sc-101199), and YAP (sc-15407). The following antibodies were purchased from Cell Signaling Technologies (Danvers, MA, USA): pFAK (Y397) (#8556s), pYAP (Ser127) (#4911), YAP/TAZ (D24E4), pGSK-3α/β (#9331s), LATS1 (#3477), BIM (#2933), cleaved-Caspase-3 (#9661), and cleaved PARP (#5625). The following antibodies were purchased from Sigma-Aldrich Israel: αTubulin (T6074), Flag (M2) (F7425). The following antibody was purchased from abcam: HA-tag (ab9110). The following antibody was purchased from Novus: LATS2 (NB200-199). Polyclonal anti-PYK2 antibody was prepared as described previously^43^. Protein A/G PLUS-Agarose beads (sc-2003) were purchased from Santa Cruz Biotechnology (Santa Cruz, CA). Cyanine Cy3-conjugated goat anti-rabbit and goat anti-mouse immunoglobulin Gs (IgGs) were purchased from Jackson ImmunoResearch Laboratories (West Grove, PA, USA). Alexa-488 donkey anti-mouse and anti-rabbit IgGs were purchased from Invitrogen (Carlsbad, CA). Hoechst 33342 and PF431396 (PZ0185) were obtained from Sigma-Aldrich (Rehovot Israel). PF573228 (324878) and MG132 (474790) were purchased from Calbiochem (Merck Millipore, USA). BIO-X (361551) was purchased from Calbiochem (Merck Millipore, USA). SB216763 was a generous gift of Prof. Hagit Eldar-Finkelman (Sackler School of Medicine, Tel Aviv University, Tel Aviv, Israel).

### Cell culture

All cell lines that used in the study were originally obtained from ATCC. HCC38 was a kind gift from Maire Virginie (Institut Curie, Research Center, Paris, France; 2014). HCC1937, MDA-MB-468, HCC1143, HCC38, MDA-MB-231, BT549, SUM159, and Hs578T cells were grown in RPMI (Gibco BRL; Grand Island, NY, US). Hs578T medium was supplemented with 2 mM l-glutamine. BT20 cells were grown in Eagle’s Minimum Essential Medium (MEM-Eagle’s) supplemented with 1 mM sodium pyruvate and 2 mM l-glutamine. HEK293T cells were grown in DMEM (Gibco BRL; Grand Island, NY, US). All media preparations were supplemented with 10% fetal bovine serum (Gibco BRL, Grand Island, NY, US), and a penicillin–streptomycin mixture (100 U ml^−1^; 0.1 mg ml^−1^; Beit Haemek, IL). Cell lines were routinely checked for the presence of mycoplasma at the interval of every 2 months.

### shRNA lentivirus production and infection

Two different shRNA sequences were used to downregulate PYK2 expression. One was purchased from Sigma (TRCN00000231519), whereas the second one was described previously^44^. Lentivirus production and infection were conducted essentially as previously described^45^. The PYK2 shRNA sequence was cloned into the pLKO.1-puro lentiviral vector. HA-tagged wild-type PYK2 was subcloned into the pHAGE PGK-IRES-Hygro-W lentiviral vector. Infected cells were grown in selection medium containing 1 µg ml^−1^ (except for BT549, 2 µg ml^−1^) puromycin or 100 µg ml^−1^ hygromycin for 72 h.

### siRNA transfection

For siRNA-mediated KD SMARTpools (Dharmacon) (Table S1) were used with Dharmafect 1 transfection reagent according to the manufacturer’s instructions.

### Immunoblot analysis

Performed as described previously^45^. Briefly, cells were washed with cold phoshate-buffered saline (PBS) and lysed in cold lysis buffer (0.5% Triton-X-100, 50 mM Hepes pH 7.5, 100 mM NaCl, 1 mM MgCl_2_, 50 mM NaF, 0.5 mM NaVO_3_, 20 mM β-glycerolphosphate, 1 mM phenylmethylsulphonyl fluoride, 10 μg ml^−1^ leupeptin, and 10 μg ml^−1^ aprotinin), vortexed and incubated on ice for 15 min. Cleared cell extracts were obtained by centrifuging at 14,000 rpm for 15 min at 4 °C. Protein concentration in each sample was estimated by Bradford assay (Bio-Rad, Hercules, CA) and equal protein amounts were analyzed by SDS–polyacrylamide gel electrophoresis and WB using standard procedures. Blocking buffer containing 5% nonfat dry milk in TBS-Tween (0.05%) was used. For densitometric analysis, the intensity of protein bands was measured using the Image J software (NIH, USA).

### Immunoprecipitation

Immunoprecipitation studies were performed as described previously^28^. Briefly, cells were washed with cold PBS and lysed using cold lysis buffer described above, centrifuged at 14,000 rpm for 15 min to obtain cleared lysates. The supernatants were incubated for 3 h at 4 °C with the indicated primary antibody bound to protein A/G Sepharose beads. The beads were then washed three times with cold HNTG (20 mM HEPES pH 7.5, 150 mM NaCl, 10% Glycerol, 0.1% Triton-X-100) buffer. The samples were loaded into protein SDS–PAGE gels and subjected to Western blot analysis.

### IF staining

IF staining was performed as described previously^28^. Briefly, cells were grown on coverslips, washed with PBS and fixed in 4% paraformaldehyde for 18 min at room temperature. The fixed cells were then incubated for 15 min in PBS containing 0.1 M glycine, incubated in blocking buffer containing 0.1% Triton-X-100, 10% goat serum and 2% BSA in TBS for 30 min, followed by 1 h incubation with the primary antibody, three washes in PBS, and then 1 h incubation with the secondary antibody. After washing with PBS, the cells were incubated for 5 min with 2 μg/ml Hoechst 33342 and mounted on microscopic slides using mounting media (10 mM phosphate buffer, pH 8.0, 16.6% w/v Mowiol4–88 and 33% glycerol). The specimens were analyzed either by using a confocal laser-scanning microscope (Zeiss 510; Carl Zeiss, Jena, Germany) or by Axio Imager 2 microscope (Zeiss). Intensity of IF staining was measured using the ImageJ software (NIH, Bethesda, MD). In brief, multiple IF images (*n* > 5) were taken for each experimental setting to evaluate relative mean fluorescence intensity. Data were expressed as mean values (±SD) of three different experiments and statistical significance was evaluated using the Student’s *t* test.

### RNA extraction and real-time PCR analysis

RNA was purified using Tri Reagent (Sigma). cDNA was generated using oligo(dT) primer and M-MLV reverse transcriptase (Promega, Madison, WI, USA). Real-time PCR analysis was performed using SYBR Green I as a fluorescent dye, according to the manufacturer’s guidelines using the ABI StepOnePlus 7500 Real-time PCR system (Applied Biosystems; Invitrogen). All experiments were normalized to GAPDH RNA levels. The primer sequences are listed in Table S2.

### Cell viability

For cell viability assays, cells were plated in 96-well plates at the indicated numbers in triplicates. Cell viability was assessed after 24 and 72 h by MTT (3-(4,5-dimethylthiazolyl-2)−2,5-diphenyltetrazolium bromide) colorimetric assay. The cells were incubated with medium containing MTT solution (0.5 mg/ml; Sigma) for 3 h at 37 °C. Cells were lysed with 100 μl lysis buffer (0.4% NP-40 in 0.04 mol/l HCl-isopropanol), and absorbance was measured at 570 nm with a 680 nm reference wavelength using ELISA microplate reader (Corning, NY, US). Cell viability is depicted as fold growth at 72 h compared to 24 h. The number of seeded cells to obtain low and high densities in different TNBC cell lines were as follows: HCC1937—low: 1.5 × 10^4^ ml^−1^; high: 1 × 10^5^ ml^−1^, MDA-MB-468—low: 2 × 10^4^ ml^−1^; high: 1.6 × 10^5^ ml^−1^, Hs578T—low: 1 × 10^4^ ml^−1^; high: 7.5 × 10^4^ ml^−1^, BT549—low: 1 × 10^4^ ml^−1^; high: 7.5 × 10^4^ ml^−1^.

### Cellular apoptosis—Annexin V affinity assay

Apoptosis was measured using Annexin V-FITC apoptosis detection kit (ab14085 Abcam, Cambridge, UK) in adherent TNBC cells according to the manufacturer’s instructions. Briefly, cells were grown on glass, washed with PBS and incubated with Annexin V-FITC (1:100) and propidium iodide (PI, 10 µg/ml) in binding buffer for 10 min at room temperature. Apoptotic cells were detected by IF microscopy using a FITC and Texas Red filter on an Axio Imager 2 microscope (Zeiss). Apoptosis was quantified using ImageJ software (NIH, Maryland, USA) by calculating the ratio between Annexin V-FITC positive cells to total cell number in multiple captured fields (~300 cells/field; total 5 fields). The mean values ± SD of three replicates per treatment are shown.

### Luciferase assay

Cells were seeded to reach 70% confluency. The cells were transfected with the firefly luciferase reporter plasmid (GTIIC-TEAD) and Renilla luciferase plasmid as internal control using JetPEI (Polyplus-transfection). After 24 h, a dual luciferase assay was performed according to the manufacturers protocol (Promega). All experiments were performed in triplicates and normalized to *Renilla* luciferase activity. Luminescence was read in black 96-well plates (NUNC) with the aid of an Infinite M200 plate reader (TECAN).

### Reverse phase protein arrays (RPPA)

RPPA was performed in the MD Anderson Cancer Center Functional Proteomics Core Facility.

http://www.mdanderson.org/education-and-research/resources-for-professionals/scientific-resources/core-facilities-and-services/functional-proteomics-rppa-core/

## Electronic supplementary material

Suppl.Legends

Figure S1

Figure S2

Figure S3

Figure S4
