# Supplementary material for: PYK2 negatively regulates the Hippo pathway in TNBC by stabilizing TAZ protein
Source: Cell Death Dis. 2018 Sep 24;9(10):985. doi: 10.1038/s41419-018-1005-z (PMC6155151; doi:10.1038/s41419-018-1005-z)
Supplement: Supplementary file 1 — Suppl.Legends [file 41419_2018_1005_MOESM1_ESM.docx]

**Supplementary legends**

**Figure S1**. PYK2 depletion attenuates growth of sparse compared to dense cells. The indicated control and PYK2-KD TNBC cell lines were seeded at increasing densities and cell viability at 24 h and 72 h was determined by MTT assay. The ratio of cell viability between 72 h and 24 h is shown (mean ±SD; n=3). Number of seeded cells (x1000) per well of 96-wells plate were: MDA-MB-468: 2, 4, 8, 16; HCC1937: 1.5, 5. 7.5, 10; BT549: 1, 2.5, 5, 7.5; Hs578T: 1, 2.5, 5, 7.5.

**Figure S2**. The protein expression levels of PYK2 and TAZ correlate positively in TNBC cell lines and in breast cancer samples. **A** WB analysis of TAZ protein level in control and PYK2-depleted TNBC cell lines using two different shRNAs or smartpool siRNAs of PYK2. Quantification is shown as fold of control. **B** PYK2 restores the level of TAZ in PYK2-depleted cells. Wild-type PYK2-HA was expressed in control and PYK2-depleted BT549 cells. The level of TAZ was assessed by WB analysis. As shown, depletion of PYK2 by two different shRNAs markedly reduced the level of TAZ protein and expression of wild-type PYK2 restored TAZ levels. **C** Scattered plot showing a Spearman correlation (Spearman coefficient = 0.576, P=2.01e-03) of PYK2 and TAZ proteins in 27 TCGA breast cancer patients. For the analysis, the TCGA_Breast_BI_Proteome.itraq.tsv (last modified: 25-Jul-2017) file was downloaded from:

https://cptcxfer.uis.georgetown.edu/publicData/Phase_II_Data/TCGA_Breast_Cancer/TCGA_Breast_BI_Proteome_CDAP_Protein_Report.r3/. The data containing global proteome data for 105 TCGA breast cancer patients was obtained using iTRAQ (isobaric Tags for Relative and Absolute Quantification; Weise et al., 2007) and proteins (PYK2 and TAZ) were quantified using the standard method described in Cancer Genome Atlas Network, 2012; https://cptac-data-portal.georgetown.edu/cptac/s/S015). Out of 105 breast cancer patient samples only 27 common patients had protein expression data (log ratio) for both PTK2B and TAZ. Spearman correlation was done between the protein expression of PTK2B and TAZ for these 27 patients. Results of correlation in protein expression were presented in a scattered plot graph.

**Figure S3.** Effect of PYK2 on TAZ protein stability is partially mediated by GSK-3β and LATS1/2. **A**, **B**, **C** Control and PYK2-depleted TNBC cell lines were treated with the indicated GSK-3β inhibitors (LiCl 10mM, BIO-X 2 μM, SB216763 5 μM) for 16 h prior to Western blot analysis to detect the level of TAZ protein. As shown, inhibition of GSK-3β only partially restored the level of TAZ. **D.** Co-inhibition of LATS1/2 and GSK-3β almost restored the level of TAZ in PYK2-depleted MDA-468 (80%). The effect varied between TNBC cell lines.

**Figure S4.** PYK2 and YAP/TAZ affect cell apoptosis. **A** Protein expression of BIM in control and PYK2-KD TNBC cell lines as quantitated by RPPA analysis data shown is fold change of control in normalized Log2 values. **B, C** WB analysis of control and either YAP/TAZ-KD (B) or PYK2-KD (C) HCC1937 and MDA-468 cells for cleaved PARP protein.

**Table S1:**

| SMARTpools (Dharmacon) | | |
| --- | --- | --- |
| siRNA name | RefSeq no. | Sequence |
| LATS1 | M-004632-00 | (1) GAACCAAACUCUCAAACAA  (2) GCAAGUCACUCUGCUAAUU  (3) GAAAUCAAGUCGCUCAUGU  (4) GAUAAAGACACUAGGAAUA |
| LATS2 | M-003865-02 | (1) GUUCGGACCUUAUCAGAAA  (2) GAAAGAGUCUAAUUACAAC  (3) GAUCGGUGCCUUUGGAGAA  (4) GAACGAUGCCAGCGAAGGU |
| YAP1 | M-012200-00 | (1) GGUCAGAGAUACUUCUUAA  (2) CCACCAAGCUAGAUAAAGA  (3) GAACAUAGAAGGAGAGGAG  (4) GCACCUAUCACUCUCGAGA |
| TAZ | M-016083-00 | (1) GACAUGAGAUCCAUCACUA  (2) GGACAAACACCCAUGAACA  (3) AGGAACAAACGUUGACUUA  (4) AAGCCUAGCUCGUGGCGGA |

**Table S2:**

| Primer sequences for qRT-PCR | |
| --- | --- |
| PYK2-Forward | CTCATCGGGAGCGTGGAT |
| PYK2-Reverse | GCTCTGCCAGGTCTTTGTTGA |
| TAZ-Forward | CGATGACCCCAGACATGAGA |
| TAZ-Reverse | CTCGAATGATATGGCCCTCC |
| CYR61-Forward | CACACCAAGGGGCTGGAATG |
| CYR61-Reverse | CCCGTTTTGGTAGATTCTGG |
| CTGF-Forward | GCAGAGCCGCCTGTGCATGG |
| CTGF-Reverse | GGTATGTCTTCATGCTGG |
| BIM-Forward | GCCAGCCCTGGCCCTTTTGC |
| BIM-Reverse | TGGGCGATCCATATCTCTGGGCG |
| GAPDH-Forward | GACAGTCAGCCGCATCTTC |
| GAPDH-Reverse | CGTTGACTCCGACCTTCAC |
