# Supplementary figures and images for: PYK2 negatively regulates the Hippo pathway in TNBC by stabilizing TAZ protein
Source: Cell Death Dis. 2018 Sep 24;9(10):985. doi: 10.1038/s41419-018-1005-z (PMC6155151; doi:10.1038/s41419-018-1005-z)

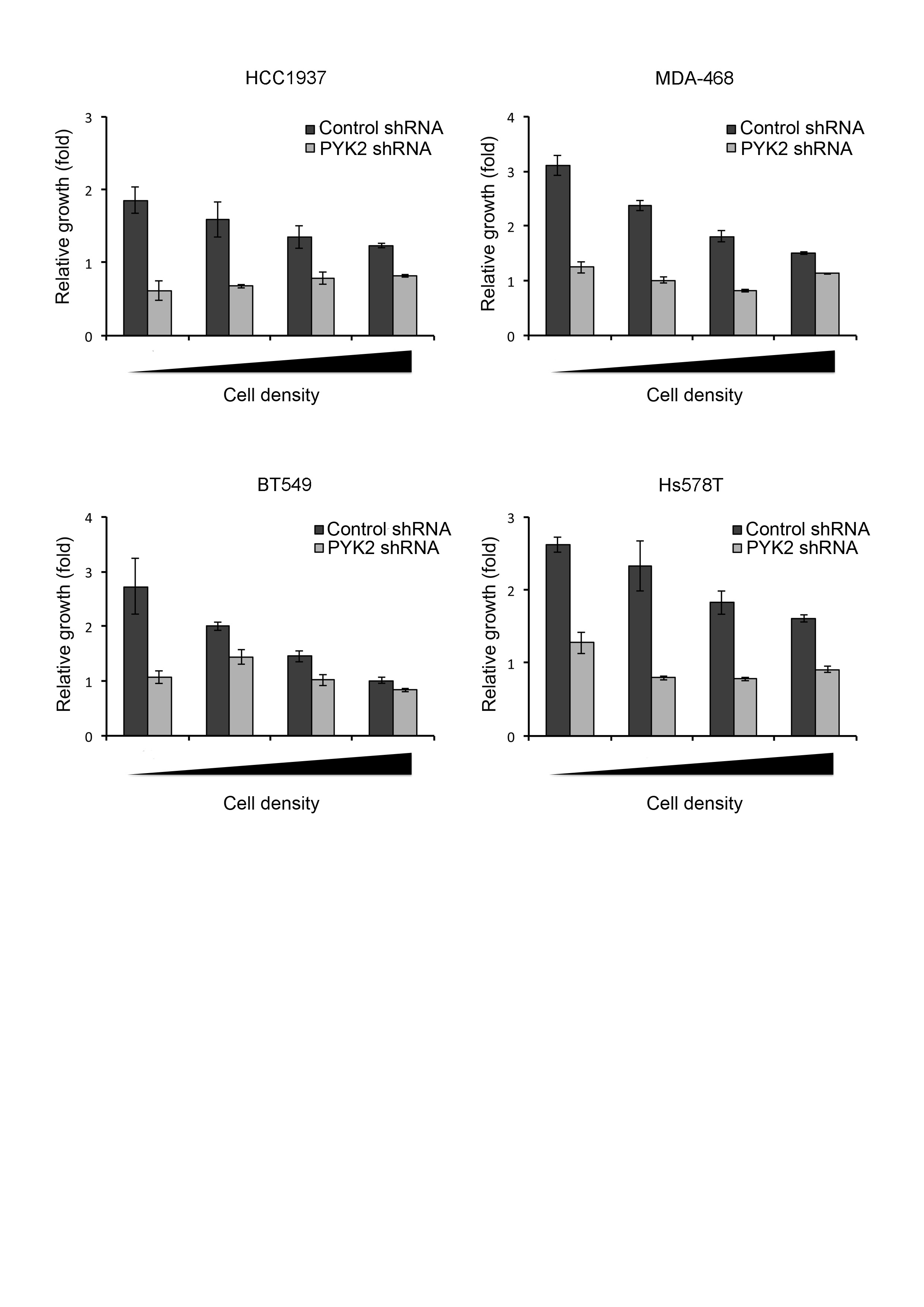

Supplement: Supplementary file 2 — Figure S1 [file 41419_2018_1005_MOESM2_ESM.jpg]

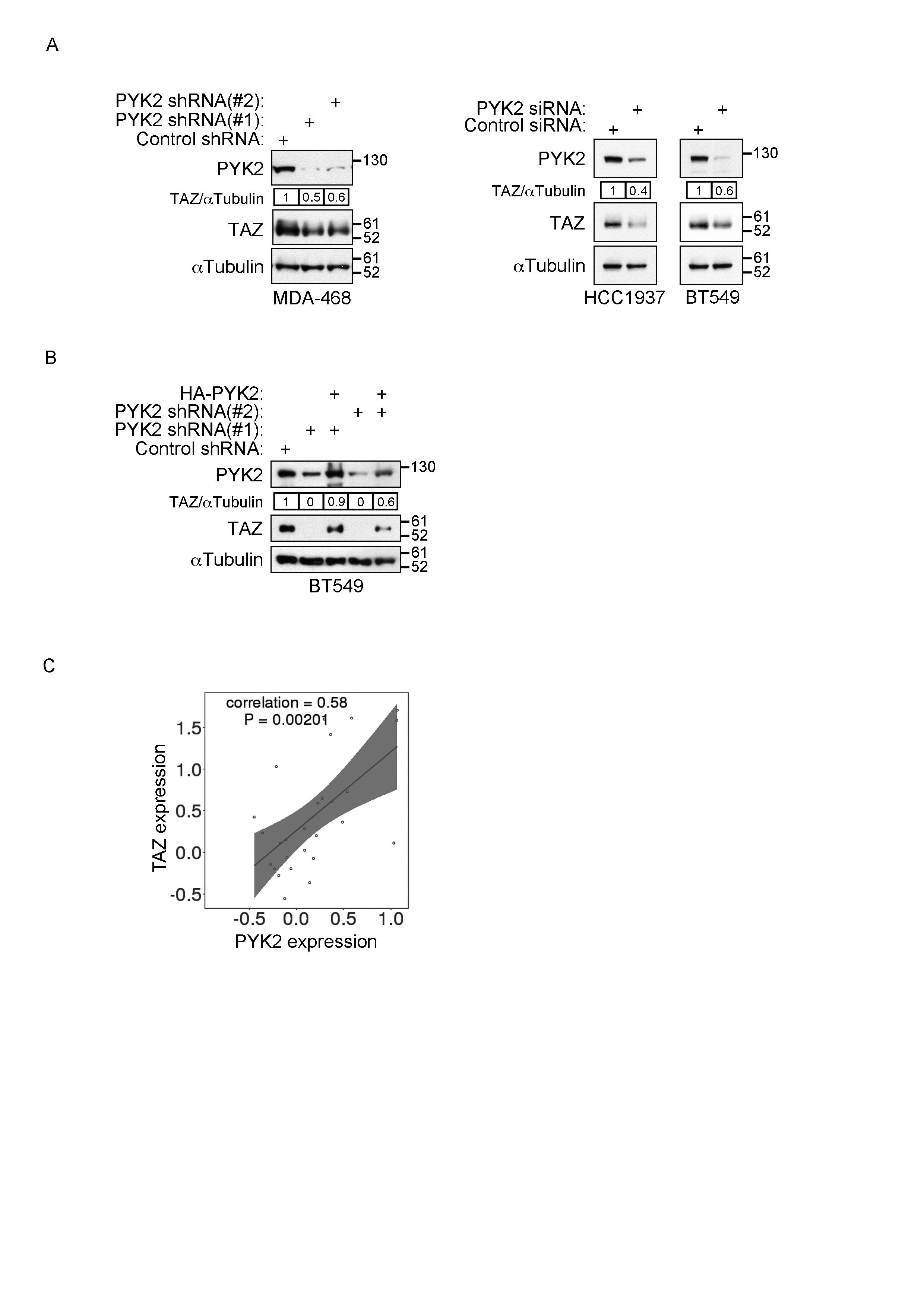

Supplement: Supplementary file 3 — Figure S2 [file 41419_2018_1005_MOESM3_ESM.jpg]

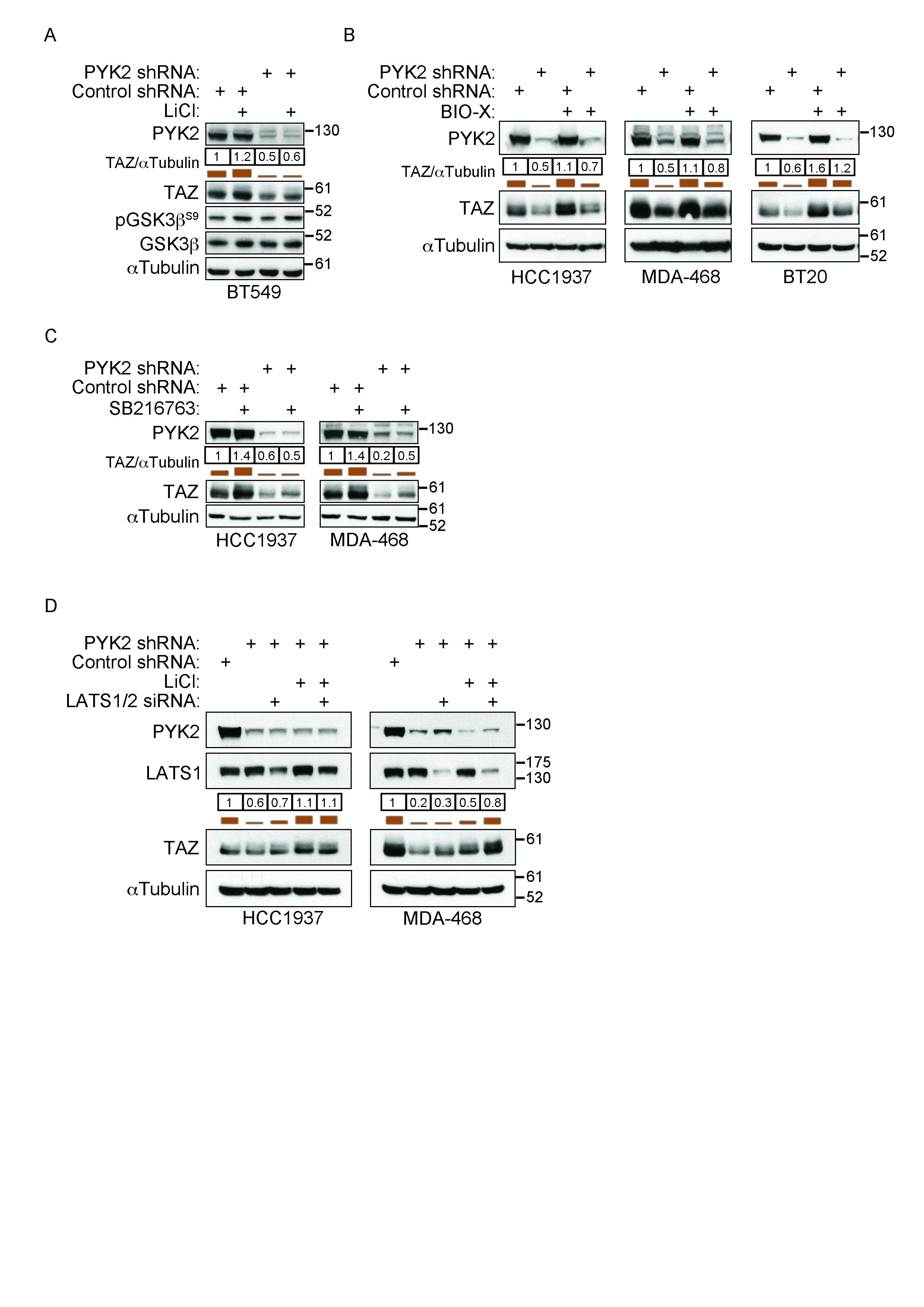

Supplement: Supplementary file 4 — Figure S3 [file 41419_2018_1005_MOESM4_ESM.jpg]

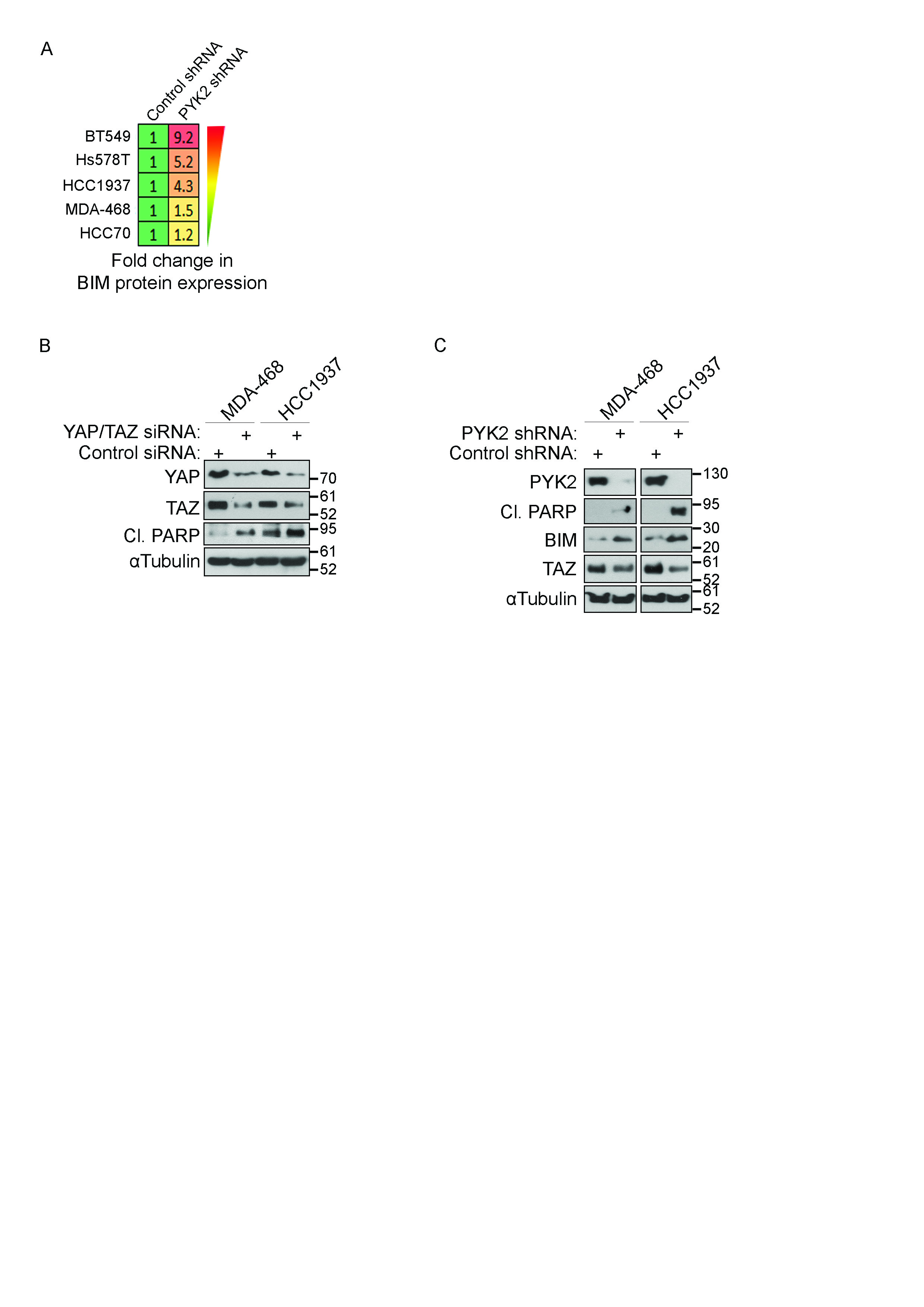

Supplement: Supplementary file 5 — Figure S4 [file 41419_2018_1005_MOESM5_ESM.jpg]
